# *Leishmania donovani* Activates Hypoxia Inducible Factor-1α and miR-210 for Survival in Macrophages by Downregulation of NF-κB Mediated Pro-inflammatory Immune Response

**DOI:** 10.3389/fmicb.2018.00385

**Published:** 2018-03-08

**Authors:** Vinod Kumar, Ajay Kumar, Sushmita Das, Ashish Kumar, Kumar Abhishek, Sudha Verma, Abhishek Mandal, Rakesh K. Singh, Pradeep Das

**Affiliations:** ^1^Division of Molecular Biology, Rajendra Memorial Research Institute of Medical Sciences (ICMR), Patna, India; ^2^Department of Microbiology, All India Institute of Medical Sciences, Patna, India; ^3^Department of Biochemistry, Institute of Science, Banaras Hindu University, Varanasi, India

**Keywords:** HIF-1α, miR-210, NF-κB, cytokines, visceral leishmaniasis, macrophages

## Abstract

Micro RNAs (miRNAs) have emerged as a critical regulator of several biological processes in both animals and plants. They have also been associated with regulation of immune responses in many human diseases during recent years. Visceral leishmaniasis (VL) is the most severe form of leishmaniasis, which is characterized by impairment of both innate and adaptive immune responses. In the present study, we observed that *Leishmania* establishes hypoxic environment in host macrophages that induces the expression of hypoxia inducible factor-1α (HIF-1α) and miRNA-210. Further, the expression of miRNA-210 was found to be dependent on activation of HIF-1α expression. The HIF-1α silencing by siRNA resulted in significantly (*p* < 0.001) decreased expression of miR-210 in parasites infected macrophages. We also observed that in siHIF-1α or antagomir-210 treated *L. donovani* infected macrophages, the parasitic load and percentage infectivity were significantly (*p* < 0.001) decreased. Furthermore, we found that inhibition of miR-210 leads to activation of NF-κB subunit p50, and it forms heterodimer with p65 and translocates into the nucleus from the cytoplasm. This significantly (*p* < 0.05) induced the transcription of pro-inflammatory cytokines genes such as TNF-α and IL-12 in miRNA-210 inhibited macrophages compared to uninhibited macrophages whereas the level of IL-10, an anti-inflammatory cytokine, was found to be significantly decreased (*p* < 0.001). These findings suggested that *L. donovani* infection induces hypoxic environment inside the macrophages that activates HIF-1α. Further, HIF-1α upregulates miR-210, which eventually establishes a suitable environment for the survival of parasite inside the host macrophages by downregulating NF-κB mediated pro-inflammatory immune responses.

## Introduction

In 1993, a new era in basic research was started after the discovery of micro RNAs (miRNAs) in *Caenorhabditis elegans* (Lee et al., 1993; Wightman et al., 1993). miRNAs are single stranded molecules having length 19–25 nucleotides, which mediate post-transcriptional gene silencing by pairing of bases with the untranslated region (UTR) of target genes. miRNA regulates various function of cells, such as immune response, stress response, apoptosis proliferation, and differentiation (Xiao and Rajewsky, 2009; Liston et al., 2010). They also affect transcription factors, histone acetylation or DNA methylation and a single miRNA may target more than hundreds of genes (Bartel and Chen, 2004).

Leishmaniasis is included among 13 neglected tropical parasitic diseases by the World Health Organization Tropical Disease Research (WHO TDR). The disease is prevalent in more than 98 countries and common in Indian subcontinent and East Africa. About 200,000–400,000 active cases detected each year out of which 90% of the cases are reported mainly in India, Ethiopia, Brazil, Somalia, Sudan, and South Sudan (WHO, 2017). The causative agent *Leishmania* is an obligate intracellular protozoan parasite, which is transmitted by bite of different species of infected sand fly to the mammalian host. Traditionally, the disease has three clinical forms, i.e., visceral leishmaniasis (VL), cutaneous leishmaniasis (CL), and mucosal leishmaniasis (MCL). Visceral disease is the most common in Indian subcontinent and fatal if not treated (Singh et al., 2006). Approximately, 0.1 million cases of VL are estimated to occur annually in India and of these, the state of Bihar accounts for more than 70% of the cases (National Vector Borne Disease Control Programme [NVBDCP], 2014).

Hypoxia inducible factor-1α (HIF-1α) is a transcription factor activated under hypoxic condition that regulates all cellular responses to hypoxia and ensures optimal functional, metabolic, and vascular adaptation to O_2_ shortages (Semenza, 2011). It is widely expressed in innate and adaptive immune responses regulating cell populations including macrophages and lymphocytes (Cramer et al., 2003; Walmsley et al., 2005; Jantsch et al., 2008; McNamee et al., 2013). Its activation was reported as a general phenomenon in infections with human pathogens (Werth et al., 2010). In CL, the role of HIF-1α has been reported where it helps in the survival of parasites by stabilizing the hypoxic environment (Arrais-Silva et al., 2005; Degrossoli et al., 2011; Schatz et al., 2016). In addition, it has been documented that hypoxia controls the inflammatory response in human dendritic cells after *Leishmania* infection (Bosseto et al., 2010). Further, drugs like echinomycin and resveratrol have shown to target HIF-1α to reduce the growth of *L. amazonensis* (Dal’Bó Pelegrini et al., 2016).

NF-κB transcription factor family comprises five members, i.e., p65 (Rel A), Rel B, c-Rel, p50, and p52 that functions as homo and heterodimers. NF-κB is normally remained as inactive in the cytoplasm by IkB. After activation, IkB is phosphorylated by IKK that degrades IkB resulting in the release of the NF-κB. After release, it translocates into the nucleus where it activates the transcription of various pro-inflammatory cytokines by binding to their promoter region (Senftleben et al., 2001; Lawrence, 2009). Studies have demonstrated a cross signaling between the HIF-1α and NF-κB (D’Ignazio and Rocha, 2016) but direct linkage between these two molecules has yet to be investigated. Since HIF-1α is a macrophage associated factor and the parasite *Leishmania* resides in the host macrophages, we planned this study to elucidate the role of HIF-1α in parasite survival.

The increasing data suggested that several classes of pathogens can manipulate the miRNA networking system of affected cells which are key regulators of host immune response and disease pathogenesis. For example, miR-155 creates suitable immunological environments for the survival of *Mycobacterium tuberculosis* inside the host macrophages and also works as a marker of the disease (Wu et al., 2012). Let-7 families of miRNAs are shown to act as first line of defense against *Salmonella typhimurium* in the host (Schulte et al., 2011). In the protozoan infection such as *Cryptosporidium parvum*, miR-221, and miR-27b have been found to play significant role in disease pathogenesis (Gong et al., 2011). In *Leishmania donovani* infection, miR-30A-5P has been shown to play a significant role in regulation of autophagy (Singh et al., 2016). In addition, upregulation of miR-294 and 721 modulates the nitric oxide synthase 2 (NOS2) and L-arginine in *Leishmania amazonensis* infection in the favor of parasite survival (Muxel et al., 2017).

HypoxamiRs (miR-24-1, miR-26, miR-103, miR-181, miR-213, and miR-210) are a class of miRNAs, which are activated under low oxygen tension and induced by HIF-1α. miRNA-210 is one of the first hypoxamiR reported, which is targeted by HIF-1α (Kulshreshtha et al., 2007). Emerging data have also proved the role of miR-210 in various human diseases (Zhang et al., 2012; Grosso et al., 2013) and also found to act as a regulator of immune responses (Qi et al., 2012; Zhang et al., 2012; Zhao et al., 2014). In *Leishmania* infection, the manifestation of the disease occurs through impairment of host immune responses, and in our previous study, we found that miR-210 is greatly upregulated in macrophages during *L. donovani* infection (Tiwari et al., 2017). We further hypothesized that the hypoxic conditions inside the host macrophages may regulate inflammatory cytokines production through HIF-1α induced miR-210/NF-κB mediated mechanisms. Herein, we provide a clue that HIF-1α controls the NF-κB mediated regulatory pathways of macrophages by upregulating miR-210 expression and helps in survival of parasites inside the host.

## Materials and Methods

### Ethics Statement

This study was carried out in accordance with the recommendations of “Institutional Animal Ethical Committee” of the Rajendra Memorial Research Institute of Medical Sciences (Patna, India). The protocol was also approved by the “Institutional Animal Ethical Committee” of the Rajendra Memorial Research Institute of Medical Sciences (Patna, India, Ref No. 364/GO/R/S/2001/CPCSEA).

### Animals

Female BALB/c mice of age 6–8 weeks were used in this study. The animals were kept in polypropylene cages and bedding material was chopped wheat straw with the temperature around 20–30°C. All the experimental mice were fed a standard chow and water *ad libitum*.

### Culture of *L*. *donovani*

A cloned line of *L. donovani* strain (AG83) was used in this study. The motile form of promastigotes was cultured in complete Dulbecco’s Modified Eagle Medium (DMEM, pH 7.2; Gibco, United States), which contained 10% heat-inactivated fetal bovine serum (FBS; Gibco, United States), 2 mM L-glutamine, sodium bicarbonate (3.7 gm/L), penicillin (100 U/mL), streptomycin (100 μg/mL), and gentamicin (20 μg/mL) at 26°C in a BOD incubator. In all stages of experiments, metacyclic promastigotes were used and obtained from late log phage to stationary stage using standard protocol (Späth and Beverley, 2001).

### Isolation of Macrophages (Mφ) From Mice

A 4% starch solution was injected to peritoneal cavity of mice and after 48 h macrophages were isolated by standard protocol. Cells were thoroughly washed and suspended in RPMI medium supplemented with 10% FBS and antibiotics. Further, macrophages (10^6^ cells per well) were plated in six well culture plates and incubated in CO_2_ incubator in 5% CO_2_ atmosphere. After overnight incubation, the non-adherent cells were removed and fresh medium was added to each wells.

### Infection of *L. donovani* in Macrophages

The macrophages were infected with *Leishmania* parasites for 6 h at a cells/parasite ratio of 1:10. After 6 h, the unbound parasites were removed by washing with RPMI without FBS and incubated up to 24 h as per our experimental conditions. The cells were then fixed with formaldehyde followed by staining with May-Grünwald Giemsa and observation under bright field microscope at 100× with oil immersion objective.

### Measurement of HIF-1α Expression

We evaluated the expression of HIF-1α mRNA in *L. donovani* infected and uninfected macrophages by semi-quantitative PCR and further validated by real-time PCR (ABI 7500, Applied Biosystem) using HIF-1α specific primers. The cycling conditions for qPCR were as follows: 1 cycle at 95°C for 3 min and 40 cycles of 95°C for 15 s (denaturation), 56°C for 30 s (annealing), and 72°C for 30 s (extension). Results are the target/reference ratios of each sample, normalized by the target/reference ratio of the calibrator. Here, the target/reference values of uninfected macrophages were used as the calibrator and the GAPDH was used as the reference.

### Measurement of Cellular Hypoxia

Pimonidazole is a novel marker for confirmation of cellular hypoxia (Varia et al., 1998). The cellular hypoxia was estimated by detection of pimonidazole adducts by western blotting using Hypoxyprobe^TM^-1 kit (Hypoxyprobe-1, Chemicon). Briefly, 250 μM of pimonidazole hydrochloride was added to *L. donovani* infected and uninfected macrophage culture. After 24 h, proteins were isolated from macrophages and quantified by Lowry’s method (Lowry et al., 1951). The protein (40 μg/lane) was then subjected to SDS–PAGE and transferred on to the nitrocellulose membrane. Further, protein bands on membrane were blocked by TBST buffer with [5% (w/v) non-fat dry milk containing 0.1% (v/v) Tween20] for 2 h. After three times washing the membrane with TBST buffer, membrane was incubated with mouse anti-pimonidazole antibody diluted 1:50 (Chemicon) for 2 h at room temperature (RT). After routine wash with TBST, membrane was incubated with horseradish peroxidase-conjugated secondary antibody, diluted in the ratio of 1:400 for 2 h. Protein bands on the blot were developed by 0.03% (v/v) DAB, 0.01% (v/v) H_2_O_2_ solution. β-actin was used as house-keeping protein.

### Next-Generation Sequencing, Real-Time Validation, and Selection of miRNA

Small RNA library preparation, TruSeq small RNA library preparation, and miRNAs’ expression are reported in our previous study (Tiwari et al., 2017). In this study, we selected differentially expressed upregulated and downregulated miRNAs for real-time validation. For real-time validation, total RNA was isolated using TRIzol method and quantified by Nanodrop spectrophotometer and cDNA was prepared using miRNA specific primers and following the standard protocol. Briefly, 1 μg of total RNA was taken and reverse transcribed using 20U M-MLV reverse transcriptase (Fermentas, Germany), 1× RT buffer, 20 mM dNTPs (New England Biolabs, United States), 20U RNasin (Fermentas, Germany), 100 ng of random hexamers (Fermentas, Germany), 0.1 M DTT, and DEPC treated water. The expression level was quantified on ABI 7500 Fast system as per manufacturer instructions (Applied Biosystem) using 5 pmol/μL of each miRNA specific primer. Briefly, 20 μL of real-time mixture was prepared which contained 1 μL cDNA, 6 μL MilliQ water, 10 μL of Power SYBER green master mix (Applied Biosystem), and 1.5 μL of forward and reverse primers. Temperatures’ setting for PCR was as follows: initial incubation of 50°C for 2 min, denaturation at 95°C for 10 min and 40 cycles at 95°C for 15 s, 60°C for 1 min, and 72°C for 40 s. The comparative *C*t method (ΔΔCt) was used to determine the level of expression of miRNA. The calibrator used for miRNA analysis was non-infected macrophage. Sno miRNA-202 was used as endogenous controls for expression analysis (Choi et al., 2013). We were calculated the fold change (2^-ΔΔ^*^C^*^t^) using the formula normalized gene expression: (2^-Δ^*^C^*^t^) in the infected macrophage divided the normalized gene expression (2^-Δ^*^C^*^t^) in the uninfected macrophages as described elsewhere (Livak and Schmittgen, 2001).

### Silencing of HIF-1α Gene

For evaluating the role of HIF-1α in regulation of miR-210 expression in *L. donovani* infected macrophage, HIF-1α was silenced by HIF-1α specific siRNAs or scramble control (Santa Cruz Biotechnology, United States). The silencing of HIF-1α was done using the company specific protocol and reagents. Briefly, macrophages (10^6^ cells/well) were re-suspended in 500 μL transfection medium and transfected with 25 nM siRNA or scramble control. After 6 h of incubation, an additional 500 μL RPMI 1640 complete medium was added and cells were cultured overnight in a six-well culture plate. Next day, cells were washed and further experiments were executed. Silencing efficiency of HIF-1α was evaluated by semi-quantitative PCR. β-actin gene was used as loading control. Further, expression of miR-210 in HIF-1α silenced macrophages was evaluated by qPCR using the miRNA-210 specific primers as described earlier. Sno202 miRNA was taken as an endogenous control.

### Bioinformatics Analysis and Luciferase Reporter Assay

We predicted the target of miR-210 by online software miRDB and Target Scan based on target rank and score. Top 20 genes were functionally correlated with activation of NF-κB. Binding site of miR-210 and TNF receptor family was detected by Target Scan 6.0. The 3′-UTR of genes of interest containing the putative *miRNA* target site(s) WT-TNF-α receptor family (wild-type) and Mut-TNF-α receptor family (mutant) was cloned into the *Xba*I site of the pGL3 control vector containing firefly luciferase gene linked to the 3′-UTR of gene (Promega, United States). Macrophages were transfected by constructed pGL3 vector with either controls or mimics of miR-210 using Lipofectamine 2000 (Invitrogen, United States). After 24 h, cells were lyzed and luciferase activity was measured by Dual-Glo luciferase Assay System (Promega, United States) according to manufacturer’s instruction on luminometer. Firefly luciferase reading was normalized by renilla luciferase reporter.

### Evaluation of the Role of miRNA-210 in NF-κB Mediated Pro-inflammatory Immune Responses

The role of miR-210 in NF-κB mediated pro-inflammatory immune responses was evaluated by silencing with antagomir. The miR-210 antagomir (designed in our laboratory and was synthesized by Integrated DNA Technologies, New Delhi) using sequence, CAGUGUGCGGUGGGCAGUGGCU with the following modification. The 2′-OMe modified bases (2′-hydroxyl of the ribose was replaced with a methoxy group), phosphorothioate (phosphodiester linkages were changed to phosphorothioate) on the first two and last four bases, and an addition of cholesterol motif at 3′ end through a hydroxyproline modified linkage. Antagomir scramble (mismatched miR-210) was also obtained from the same company and used as negative control. The expression of miR-210 was silenced by antagomir according to the standard protocol described previously with required modification (Krützfeldt et al., 2005; Haftmann et al., 2015). Briefly, macrophages (10^6^ cells/well) were suspended in 500 μL of serum-free media for 2 h at 37°C and 5% CO_2_ supplemented with 1 μM of antagomir-210 or antagomir scramble. After incubation, 150 μL of media containing serum and antibiotics were added and culture for 24 h in CO_2_ incubator. For evaluating the functional role of miR-210, four experimental groups were plotted as follows: (1) uninfected Mφ, (2) Mφ infected with *L. donovani*, (3) miR-210 silenced Mφ infected with *L. donovani*, and (4) antagomir scramble treated Mφ infected with *L. donovani*. Macrophages were incubated in CO_2_ incubator with 5% CO_2_ for 24 h. After 24 h of incubation, supernatants were collected for measurement of cytokines, NO*_x_* and reactive oxygen species (ROS).

In another experiment to the study of the role of HIF-1α and miR-210 in parasite infectivity and survival inside the macrophages, parasitic load was measured in siHIF-1α and antagomir treated *L. donovani* infected macrophages along with negative control at 6, 12, and 24 h of infection. Percent infectivity was calculated by counting the number of infected cells and parasite load was determined by counting the number of amastigotes per 100 macrophages. The results were expressed as mean ± standard deviation (*SD*) and performed in quadruplicate.

### NF-κB Transcription Factor (p50) Activation Assay

Macrophages (10^6^ cells/well) were harvested from culture plates, centrifuged, and pellets were suspended in 1 mL cold PBS. The suspension was further centrifuged at 6000 rpm for 5 min in a cold room and supernatant was removed. Tubes were immediately kept on ice and 5X cytoplasmic proteins extraction buffer (1 M HEPES, 5 M NaCl, 0.5 M EDTA, 100% glycerol, and protease inhibitor) was added and incubated on ice with occasional vortexing from time to time. The suspension was centrifuged at 3000 rpm for 5 min and the supernatant containing cytoplasmic proteins was collected.

Prior to nuclear protein extraction, pellets obtained after cytoplasmic protein were washed with 100 μL of cytoplasmic buffer for four to five times by repeated centrifugation at 3000 rpm for 5 min. Further, 100 μL nuclear protein extraction buffer (1 M HEPES, 2 M KCl, 0.5 M EDTA, 100% NP-40, and protease) was added to the pellet and incubated on ice for 10 min with vortexing occasionally time to time. Samples were further centrifuged at 14,000 rpm for 5 min at 4°C and the supernatant containing nuclear protein was collected. The proteins were quantified and stored at -80°C for execute of further experiments. NF-κB transcription factor (p50) activation assay in cytoplasmic and nuclear proteins extract of all experimental groups was performed using NF-κB activation assay kit (Abcam, ab207217) as per protocol provided by the manufacturer. Optical density was determined at 450 nm using a spectrophotometer.

### Expression of NF-κB Transcription Factor (p50)

Expression of NF-κB transcription factor (p50 and p65) was studied by western blot in both cytoplasmic and nuclear proteins of macrophages. Equal amount of protein (40 μg/lane) was loaded and separated on 12% sodium dodecyl sulfate-polyacrylamide gel electrophoresis (SDS–PAGE). Total separated protein bands were transferred on nitrocellulose membrane (Millipore, United States). The membranes containing protein bands were blocked in 1× TBST buffer (5% (w/v) non-fat dry milk containing 0.1% (v/v) Tween20) for 1 h at RT. After blocking, membranes were washed with TBST for three times and further incubated with p50 and p65 specific primary antibodies (Santa Cruz Biotechnology, United States) for 4 h (1:200 dilutions). After incubation, membranes were washed with TBST and incubated with horseradish peroxidase-conjugated secondary antibody, diluted in the ratio of 1:500 for 2 h. Protein bands on the blots were developed by 0.03% (v/v) DAB, 0.01% (v/v) H_2_O_2_ solution. Lamin B and β-actin (Santa Cruz Biotechnology, United States) proteins were used as reference for nuclear and cytoplasmic protein, respectively. Reference proteins were used to analyze the relative band intensity by Image Analysis Software of gel documentation system (Bio-Rad). The data were represented as mean ±*SD* of band density ratio of the experiments.

### Estimation of Cytokines

The cytokine levels (TNF-α, IL-12, and IL-10) in cell culture supernatant of all the experimental groups were measured by MAX^TM^ standard set enzyme-linked immunosorbent assay (ELISA) kit as per manufacturer’s instructions (BioLegend, United States). The results were represented in pg/mL.

### Analysis of Cytokines Expression by RT-PCR

Total RNA was isolated from macrophages of all experimental groups using TRI Reagent^®^ (Sigma Chemicals, United States) following manufacturer’s instruction. Briefly, cells were pelleted by centrifugation at 5,000 rpm and pellet was washed thrice with PBS (0.02 M, pH 7.2). Further, cells were lyzed in 300 μL of TRIzol and 120 μL chloroform. The cells were centrifuged at 10,000 rpm for 10 min. The upper aqueous layer was transferred into another centrifuge tube and twice volume of isopropanol was added. The mixture containing aqueous layer and isopropanol was centrifuged again at 10,000 rpm for 10 min and RNA pellets were collected. In the last step, RNA pellets were washed with 70% (v/v) ethanol treated with RNAse-free DNase to avoid DNA contamination. In all step of centrifugation, 4°C temperature was maintained. For analysis of cytokine mRNA expression, 20 μL cDNA was prepared following the protocol describing elsewhere. Briefly, equal amount of total RNA (1 μg) was taken and reverse transcribed using 1× RT buffer, 20 mM dNTPs (New England Biolabs, United States), 0.1 M DTT with DEPC-treated water, 200 ng of random hexamers (Fermentas, Germany), 20U RNasin (Fermentas, Germany), and 20U M-MLV reverse transcriptase (Fermentas, Germany). The cDNA was subsequently amplified using mRNA specific cytokines primers. The β-actin was used as house-keeping control gene. The 25 μL of reaction mixture was prepared containing 2 μL cDNA templates, 1× polymerase chain reaction buffer, 200 μM dNTPs, 0.5 mM MgCl2, 1U Taq DNA polymerase (New England Biolabs, United States), and 3.2 μM mice mRNA specific forward and reverse primers. Amplification was performed for 22 cycles and temperature and time of PCR were set as follows: denaturation at 94°C for 30 s, annealing at 46–55°C (depended on the Tm of the primers) for 30 s, and extension at 72°C for 30 s. Before the start of PCR cycles, initial denaturation at 94°C for 5 min and after completion of cycles, final extension for 7 min at 72°C was performed. The amplified PCR products were separated on 2% agarose gel containing ethidium bromide (0.5% w/v) and visualized under UV illumination in a gel documentation system (Bio-Rad, United States). The relative mRNA expression levels were analyzed by Image Analysis Software. List of primers used in this study is given in Supplementary Table S1.

### Measurement of Total Nitric Oxide (NO*_x_*)

In culture supernatant, total nitric oxide (NO*_x_*) was estimated by measuring the amount of nitrite using Griess reagent as described elsewhere (Ding et al., 1988). The cultured supernatants (100 μL) were incubated with Griess reagent in the ratio of 1:1 for 10 min at RT. The absorbance was measured at 540 nm. The concentration of total nitrite was determined by comparing with a standard curve plotted using sodium nitrite (50–1.56 μM) as standard and expressed in micromolar.

### Measurement of Superoxide Anion (O_2_^-^) Level

The super oxide anion content was estimated in cell culture supernatant by the method described elsewhere (Johnstan et al., 1978). The principle of this method is based on the reduction of ferricytochrome c into ferrocytochrome c in the presence of O_2_^-^ that directly correlates the level of O_2_^-^ production to the cytochrome c reduced. In brief, 2 mL reaction mixture contained 100 μL of culture supernatant and 0.05 mM ferricytochrome c in PBS. Reaction mixture was incubated for 15 min at 37°C and reactions were terminated by placing the tubes on ice. Absorbance of the supernatant fractions was measured at 550 nm. The concentration of cytochrome c reduced was determined using extinction coefficient 2.1 × 10^4^ M^-1^ cm^-1^ and expressed as nmoles of O_2_^-^ liberated per mg of protein.

### Statistical Analysis

The data were analyzed by one-way analysis of variance (ANOVA) using Student’s–Newman–Keuls test by Sigma Stat 3.5 software. The *p*-value less than 0.05 was considered to be significant. All the experiments were performed in quadruplicate and the data represented as mean ±*SD*.

## Results

### *L. donovani* Increased HIF-1α Expression Levels in Infected Macrophages

**FIGURE 1:**
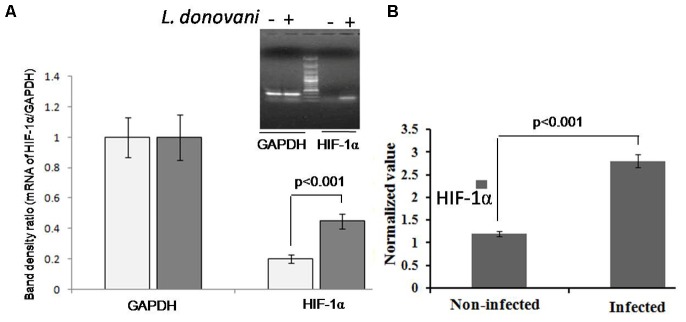
Expression of hypoxia inducible factor-1α (HIF-1α) in macrophages infected with *Leishmania donovani*. The expression of HIF-1α was evaluated by semi-quantitative **(A)** or quantitative real-time PCR **(B)**. GAPDH was taken as loading control. Each experiment was performed in quadruplicate and the values were expressed as mean ± *SD*. The level of HIF-1α in infected macrophages was significantly (*p* < 0.001) increased compared to uninfected macrophages.

*Leishmania donovani* infection in macrophages increased the expression of HIF-1α gene, which was significantly higher (*p* < 0.001) compared to uninfected macrophages. **Figure 1A** shows the band densitometry of the expression of HIF-1α mRNA and **Figure 1B** is the real-time validation in infected and uninfected macrophages.

### Confirmation of Cellular Hypoxia by Western Blotting

**FIGURE 2:**
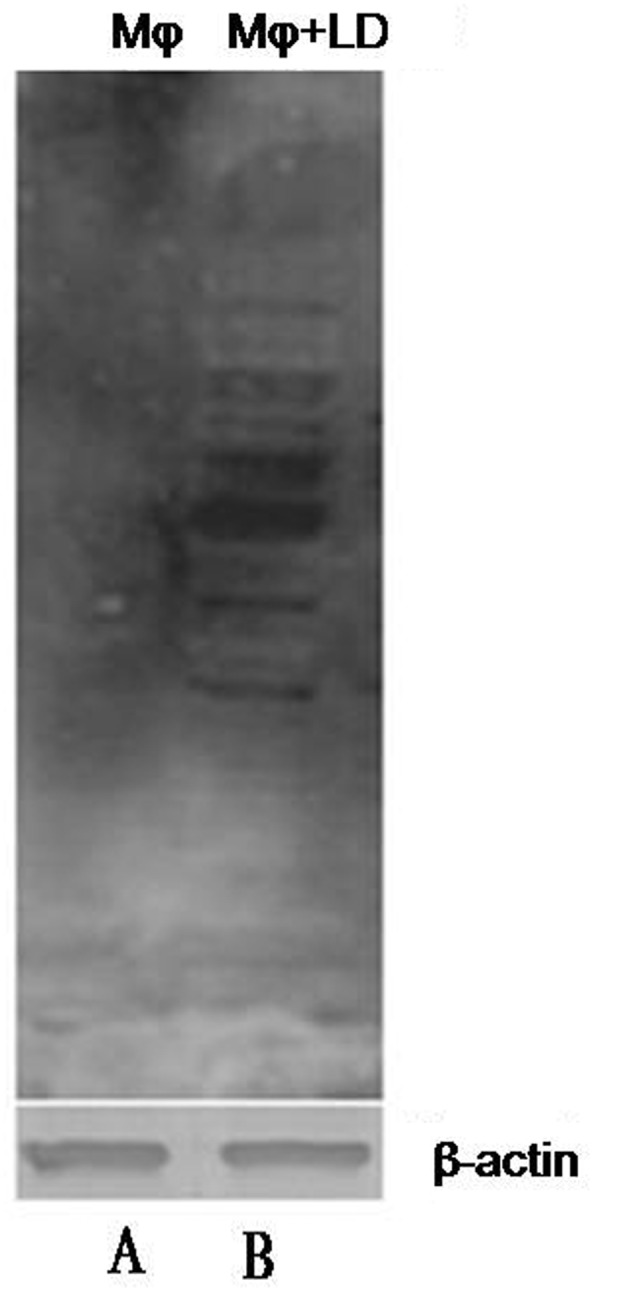
Confirmation of cellular hypoxia in *L. donovani* infected macrophages by western blotting. We detected multiple bands on blot incubated with mouse anti-pimonidazole antibody diluted 1:50 (Chemicon, United States) for 2 h at room temperature. Lane A shows only blot of macrophage protein, and Lane B shows the blot of *L. donovani* infected macrophages. The β-actin was taken as control.

Cellular hypoxia was confirmed by detecting pimonidazole adducts in protein lysate of macrophages by western blotting (**Figure 2**). In protein lysates of *L. donovani* infected macrophages, multiple bands were observed since pimonidazole forms adduct with molecules in hypoxic cells.

### Quantitative PCR for Confirmation of Differentially Expressed miRNA

**FIGURE 3:**
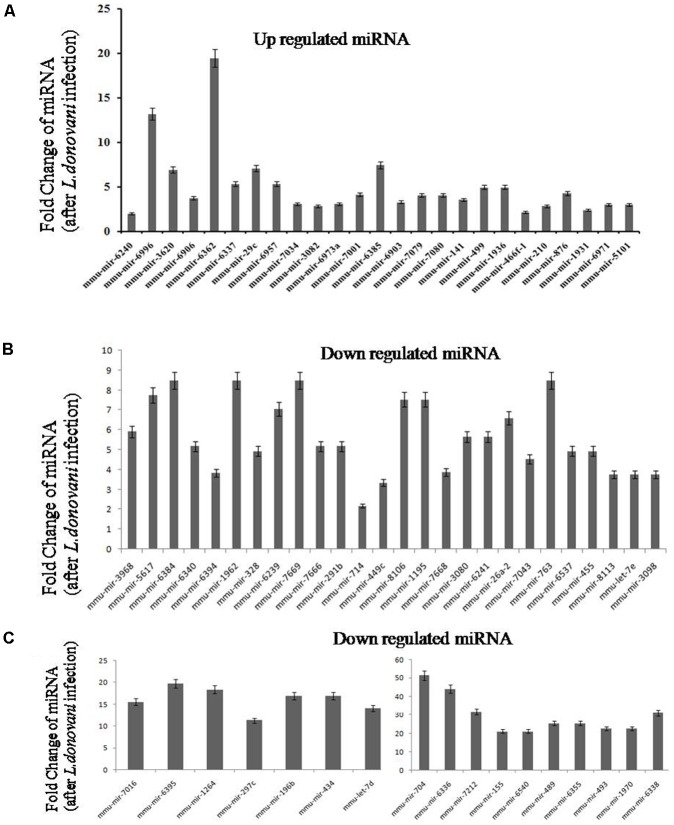
Real-time validation of upregulated and downregulated micro RNAs (miRNAs). Macrophages were infected with *L. donovani* with parasite to macrophage ratio of 10:1. After 6 h, cells were washed to remove non-ingested parasites. Infected macrophages were incubated at 37°C up to 24 h and total RNA was isolated. After next generation sequencing, 25 upregulated **(A)** and 43 downregulated **(B,C)** micro-RNA was selected for real-time validation (qPCR). The comparative *C*t method (ΔΔ*C*t) was used to determine the levels of expression for the miRNA. The calibrator used for miRNA analysis was non-infected macrophage. Sno miRNA 202 was used as an endogenous control for expression analysis. Relative quantity values or normalized levels expression were obtained by the formula 2^-ΔΔ^*^C^*^t^.

Out of 150 identified miRNA as shown in heat map (Supplementary Figure S1), we selected 25 highly upregulated and 43 downregulated miRNA for qPCR confirmation. **Tables 1**, **2** show the fold change of miRNA expression in *Leishmania* infected macrophages. Graphical representation of qPCR confirmation of differentially expressed miRNAs is shown in **Figure 3**.

**Table 1:** List of upregulated micro RNA (miRNA) in *Leishmania donovani* infected macrophages.

Name of miRNA	Fold change	*p*-Value
mmu-mir-6240	1.986	0.035
mmu-mir-6996	13.18	0.04
mmu-mir-3620	6.906	0.04
mmu-mir-6906	3.744	0.048
mmu-mir-6362	19.48	0.04
mmu-mir-6337	5.313	0.036
mmu-mir-29c	7.084	0.045
mmu-mir-6957	5.313	0.04
mmu-mir-7034	3.07	0.044
mmu-mir-3082	2.833	0.049
mmu-mir-6973a	3.086	0.049
mmu-mir-7001	4.132	0.04
mmu-mir-6385	7.438	0.046
mmu-mir-6903	3.276	0.048
mmu-mir-7079	4.073	0.028
mmu-mir-7080	4.073	0.0368
mmu-mir-141	3.542	0.041
mmu-mir-499	4.958	0.046
mmu-mir-1936	4.958	0.036
mmu-mir-466f-1	2.125	0.0486
mmu-mir-210	2.823	0.049
mmu-mir-876	4.25	0.036
mmu-mir-1931	2.391	0.045
mmu-mir-6971	3.011	0.03
mmu-mir-5101	3.011	0.04

**Table 2:** List of downregulated miRNA macrophages infected with *Leishmania donovani.*

Name of miRNA	Fold change	*p*-Value
mmu-mir-704	51.387	0.006
mmu-mir-6336	143.995	0.015
mmu-mir-7212	31.764	0.023
mmu-mir-3968	5.916	0.018
mmu-mir-7016	15.529	0.018
mmu-mir-5617	7.764	0.013
mmu-mir-6384	8.47	0.012
mmu-mir-6340	5.176	0.015
mmu-mir-155	21.176	0.01
mmu-mir-6540	21.176	0.006
mmu-mir-6395	19.764	0.01
mmu-mir-6394	3.82	0.023
mmu-mir-1264	18.352	0.017
mmu-mir-1962	8.47	0.013
mmu-mir-6338	31.058	0.013
mmu-mir-297c	11.294	0.01
mmu-mir-328	4.941	0.025
mmu-mir-489	25.411	0.01
mmu-mir-6355	25.411	0.009
mmu-mir-493	22.588	0.014
mmu-mir-1970	22.588	0.014
mmu-mir-6239	7.059	0.033
mmu-mir-7669	8.47	0.015
mmu-mir-7666	5.176	0.027
mmu-mir-291b	5.176	0.046
mmu-mir-714	2.186	0.039
mmu-mir-449c	3.337	0.047
mmu-mir-8106	7.529	0.044
mmu-mir-1195	7.529	0.029
mmu-mir-7668	3.882	0.0297
mmu-mir-196b	16.941	0.02
mmu-mir-434	16.941	0.039
mmu-mir-3080	5.647	0.042
mmu-mir-6241	5.647	0.04
mmu-mir-26a-2	6.588	0.014
mmu-mir-7043	4.518	0.032
mmu-mir-763	8.47	0.042
mmu-let-7d	14.117	0.034
mmu-mir-6537	4.941	0.037
mmu-mir-455	4.941	0.035
mmu-mir-8113	3.765	0.044
mmu-let-7e	3.765	0.036
mmu-mir-3098	3.765	0.043

### Upregulation of miRNA-210 in *Leishmania* Infected Macrophage Was HIF-1α Dependent

**FIGURE 4:**
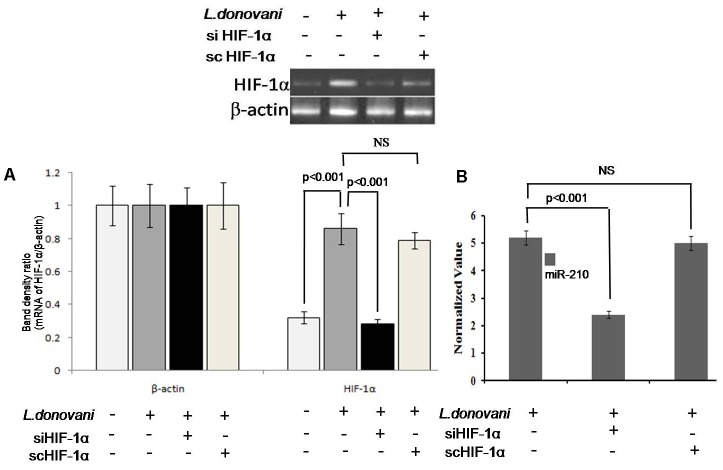
Expression of miR-210 and HIF-1α in infected macrophages. Four different experimental conditions (Mφ, Mφ+LD, siHIF-1α+Mφ+LD, and scHIF-1α+Mφ+LD) were used for expression analysis of HIF-1α by semi-quantitative PCR **(A)**. The expression of miR-210 in *L. donovani* infected macrophages in the presence of siHIF-1α or scHIF-1α was estimated by qPCR **(B)**. The sno-202 was taken as endogenous control. The application of siHIF-1α decreased (*p* < 0.001) the level of miR-210 expression in infected macrophages **(B)**.

To find out the role of HIF-1α in regulation of miRNA-210 expression, we silenced HIF-1α gene in macrophages by HIF-1α specific siRNA (Supplementary Figure S2). The HIF-1α expression was found significantly (*p* < 0.001) increased in *Leishmania* infected macrophages. However, after silencing of HIF-1α (**Figure 4A**), the expression of miR-210 was significantly (*p* < 0.001) downregulated (**Figure 4B**), which confirmed that the upregulation of miR-210 was HIF-1α dependent. Control scramble siRNA did not affect the HIF-1α either in *L. donovani* infected or uninfected macrophages.

**FIGURE 5:**
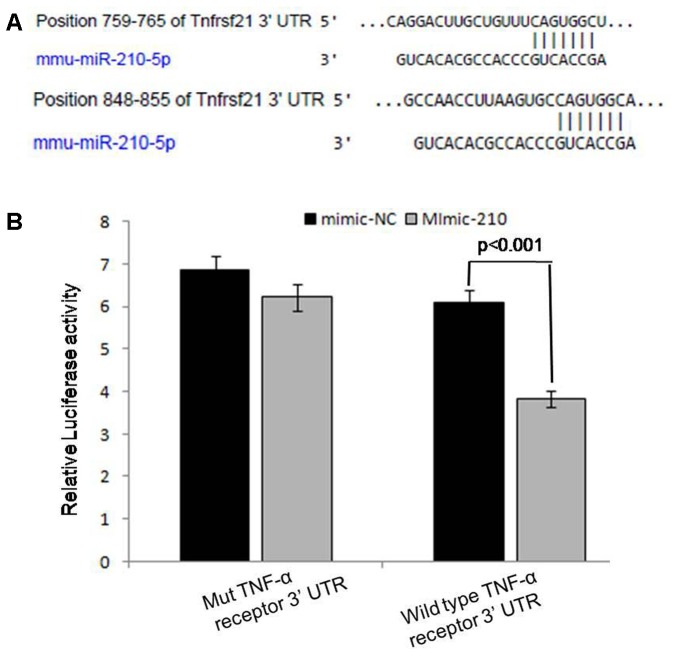
Prediction and validation of TNF-α receptor as target gene of miRNA-210. Schematic representation of miRNA-210 sequence bound to TNF-α receptor **(A)**. The luciferase constructs fused to 3′ untranslated region (UTR) (or Mut) of TNF-α receptor were co-transfected in macrophages cells with mimic-210 or mimic-NC. The luciferase activities were expressed as ratio of firefly luciferase over renilla luciferase. The macrophages transfected with mimic of miR-210 significantly reduced the luciferase activities (*p* < 0.001) of genes fused to TNF-α receptor family 3′ UTR **(B)**.

Out of top 20 targeted genes (Supplementary Figure S3) of miRNA-210, we found that sixth rank of its targeted gene, i.e., tumor necrosis factor receptor super family plays an important role in activation of NF-κB (p50) subunit by gene ontology and KEGG (data not shown) since TNF-α is the main inducer of this signaling protein. Further, for validation of miR-210 target genes, we performed the luciferase reporter assay. The transfection with mimic of miR-210 significantly (*p* < 0.001) reduced the luciferase activities of genes fused to TNF-α receptor family 3′ UTR. Mutated 3′ UTR seed sequences of TNF-α receptor family did not show any significant luciferase activities treated with mimic of miR-210 (**Figure 5**).

### HIF-1α and miR-210 Altered the Parasite Infectivity and Survival in the Macrophages

The role of HIF-1α and miR-210 was validated in the following four experiment groups, i.e., (1) uninfected Mφ, (2) Mφ infected with *L. donovani*, (3) antagomir treated Mφ infected with *L. donovani*, and (4) Antagomir scramble treated Mφ infected with *L. donovani* to validate the role of miR210 on parasitic infectivity and survival.

**FIGURE 6:**
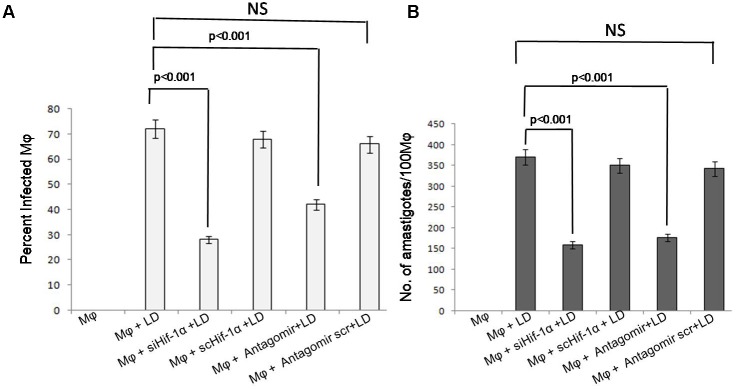
Effect of siHIF-1α and antagomir-210 on infectivity and survival of *Leishmania* parasites in the macrophages. The percentage infectivity in siHIF-1α or antagomir-210 treated macrophages was significantly (*p* < 0.001) decreased compared to untreated macrophages **(A)**. A similar pattern was also observed in parasite survival in macrophages and the amastigotes’ number per 100 macrophages was significantly (*p* < 0.001) reduced **(B)**. HIF-1α or antagomir scramble did not affect the percentage infectivity or parasite survival.

We determined the percentage of infected macrophages and parasitic load in siHIF-1α and antagomir treated macrophages with negative control. We observed that after silencing HIF-1α, parasite infectivity and parasitic load were significantly (*p* < 0.001) reduced at 24 h of infection (**Figure 6A**). A similar pattern was also observed in antagomir-210 treated macrophages where parasite infectivity and parasitic load were found to be significantly (*p* < 0.001) arrested (**Figure 6B**). Further, at 12 h of infection, parasite infectivity and parasitic load also found significantly (*p* < 0.05) decreased (Supplementary Figures S4C,D). However, after 6 h of infection, parasite infectivity and parasitic load did not show significant changes (Supplementary Figures S4A,B).

### The miR-210 Regulates the Expression of NF-κB Transcription Factor p50

**FIGURE 7:**
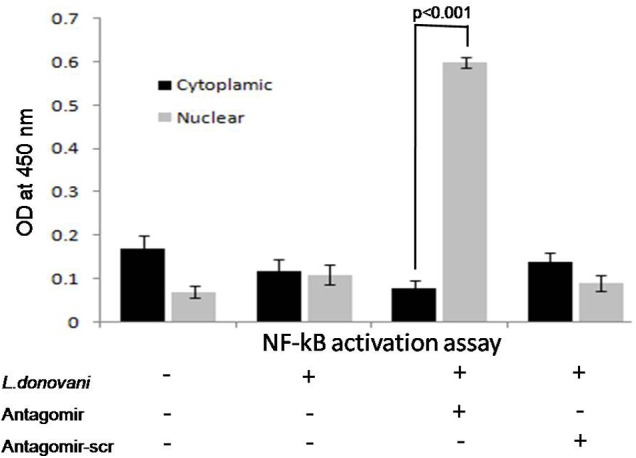
Effect of antagomir-210 in activation of NF-κB p50 subunit in cytoplasmic and nuclear protein extracts of macrophages. The optical density in nuclear protein extracts of antagomir treated macrophage was significantly (*p* < 0.001) increased. In cytoplasmic protein extract, optical density for NF-κB activation was found normal level. Antagomir scramble did not affect the NF-κB activation in either cytoplasmic or nuclear protein extracts.

The role of miR-210 in TNF-α mediated NF-κB p50 activation was investigated. In this study, we performed the NF-κB p50 activation assay in cytoplasmic and nuclear extract in protein lysates in all experimental groups. The optical density in cytoplasmic or nuclear protein lysates was not found significant before antagomir treatment. However, after treatment of macrophages with antagomir, optical density in nuclear extract protein showed optimal level and significantly (*p* < 0.001) increased (**Figure 7**).

### The miR-210 Regulates the NF-κB p50 for Pro-inflammatory Responses in *L. donovani* Infection

**FIGURE 8:**
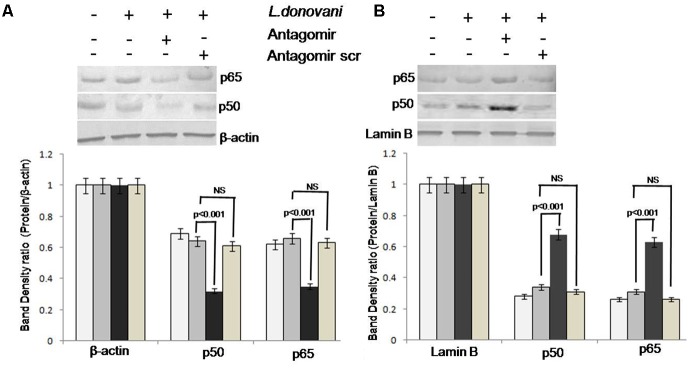
Western blot analysis of NF-κB p50 and p65 in cytoplasmic and nuclear protein extract of macrophages. Proteins were quantified by Lowry methods and equal amount of protein (40 μg/lane) was subjected to SDS–PAGE followed by western blotting using NF-κB p50 and p65 specific monoclonal antibodies. The expression of NF-κB p50 and p65 in cytoplasmic and nuclear proteins was observed by gel documentation system and the bands’ intensity was quantified by densitometry by Quantity One software. The NF-κB p50 and p65 expression in cytoplasmic protein of antagomir treated macrophages was significantly (*p* < 0.001) decreased compared to untreated macrophages **(A)**. Further, in nuclear protein, the NF-κB p50 and p65 expression in antagomir treated macrophages was significantly (*p* < 0.001) increased **(B)**. Antagomir scramble did not affect the either cytoplasmic or nuclear protein expression.

The NF-κB p50 and p65 expression in cytoplasmic and nuclear protein extracts was evaluated by western blotting (Supplementary Figures S5, S6). The expression levels of p50 and p65 in all groups of Mφ cells are depicted in **Figures 8A,B**. In cytoplasm, before and after *L. donovani* infection, p50 and p65 showed normal level of expression (lanes 1 and 2 of **Figure 8A**); however, after miR-210 silencing, the p50 protein forms heterodimer with p65 and translocated to the nucleus and its expression was significantly (*p* < 0.001) lowered in cytoplasmic protein content (lane 3 of **Figure 8A**). In a similar manner, before and after *L. donovani* infection, the expression of p50 and p65 subunit shows normal expression level in nuclear proteins (lane 1 and 2 of **Figure 8B**); however, after miR-210 silencing, the expression of p50 and p65 proteins was significantly (*p* < 0.001) higher in nuclear proteins (lane 3 of **Figure 8B**). Antagomir scramble did not affect the NF-κB p50 and p65 expression in either cytoplasmic or nuclear protein extracts. These findings clearly showed that miR-210 regulates the activation of NF-κB p50 and hypoxia induced miR-210 targets the TNF-α receptor super family gene.

### Estimation of Cytokines

**FIGURE 9:**
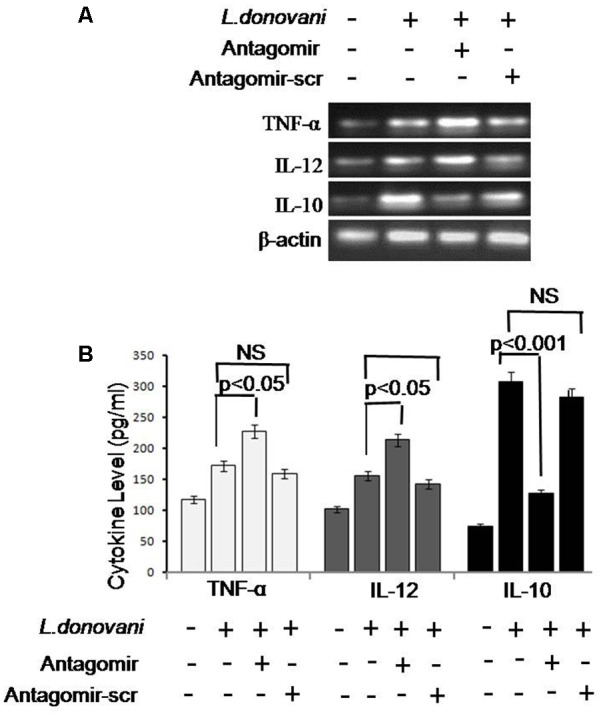
Expression of pro-inflammatory (TNF-α and IL-12) and anti-inflammatory cytokines (IL-10) cytokines. Cytokines mRNA expression levels were quantified by semi-quantitative PCR. β-actin was used as house keeping control gene **(A)**. We observed that mRNA expression of TNF-α and IL-12 was significantly upregulated after antagomir treatment; however, expression of IL-10 was significantly downregulated. Antagomir scramble did not alter the mRNA expression of cytokines. A similar pattern was also observed by enzyme-linked immunosorbent assay (ELISA) **(B)**. The infected cells expressed low levels of pro-inflammatory cytokines and increased level of IL-10. In antagomir treated macrophages, the level of pro-inflammatory cytokines was increased (*p* < 0.05) and IL-10 level was decreased (*p* < 0.001). Antagomir scramble did not affect the level of pro or anti-inflammatory cytokines.

Release of cytokines in cultured supernatant was measured by cytokine ELISA and is depicted in **Figure 9**. The level of pro-inflammatory cytokines, i.e., TNF-α and IL-12, was significantly higher (*p* < 0.05) in antagomir treated macrophages compared to untreated macrophages. However, the level of anti-inflammatory cytokines, i.e., IL-10 in antagomir treated macrophages was found lower (*p* < 0.001) compared to untreated macrophages. The level of pro-inflammatory cytokines mRNAs showed a similar pattern of result and their expression in antagomir treated macrophages was higher compared to untreated macrophages (Supplementary Figures S7–S9). The expression level of IL-10 was lower in antagomir treated macrophages compared to untreated cells. The macrophages treated with antagomir scramble did not show significant change in pro- or anti-inflammatory cytokines level compared to untreated macrophages. These results indicated that miR-210 has an important role in IL-10 cytokine production during *Leishmania* infection and its control can upregulate the production of inflammatory cytokines.

### Estimation of O_2_^-^ and NO*_x_* Level

**FIGURE 10:**
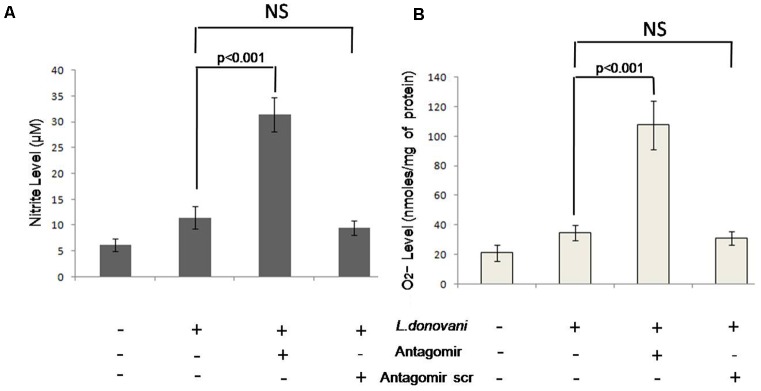
Production of nitrite and superoxide by macrophages. The level of nitrite was measured using Griess reagent and the absorbance was recorded in ELISA plate reader at 540 nm and the results were expressed in μM **(A)**. The superoxide anion absorbance was recorded at 550 nm and the results were expressed in nmoles/mg of protein **(B)**. The nitrite and O_2_^-^ levels in antagomir treated macrophages culture supernatant were significantly (*p* < 0.001) higher than non-treated groups. Antagomir scramble did not affect their level of production.

Respiratory burst activity was measured in terms of O_2_^-^ level after 2 h and NO*_x_* levels after 24 h of infection in culture supernatants and results are depicted in **Figures 10A,B**, respectively. The levels of O_2_^-^ and NO*_x_* in antagomir treated macrophages were 107.64 ± 15.26 nmoles/per mg of protein and 31.45 ± 3.12 μM, respectively. However, in untreated macrophages, their levels were 34.67 ± 5.41 nmoles/per mg and 11.53 ± 2.21 μM, respectively. The O_2_^-^ and NO*_x_* levels were found increased (*p* < 0.001) in antagomir treated macrophages compared to untreated macrophages. The antagomir scramble treated macrophages did not show significant change in either O_2_^-^ or NO*_x_* levels. These results confirmed the regulatory role of miR-210 in macrophage effector functions in terms of respiratory burst activity.

## Discussion

Low oxygen condition or hypoxia is a physiological stress that is created in affected cells in many human diseases (Semenza, 2007). After stress of low oxygen, a transcription factor HIF-1α is induced inside the affected cells and has been to shown to regulate host immune response in either the favor or the elimination of pathogens. Previously, it has been reported that after infection, several classes of pathogens such as *Salmonella typhimurium*, *Pseudomonas aeruginosa*, *Streptococcus pyogenes*, and HIF-1α promote killing of pathogens by modulating immune response of the host (Peyssonnaux et al., 2005; Nizet and Johnson, 2009). However, in *Toxoplasma gondii* infection, the activation of HIF-1α in affected host cells promotes survival and growth of this protozoan parasite albeit exact mechanisms are not known (Wiley et al., 2010). The role of HIF-1α activation and its consequences on host immune mechanisms in protozoan parasites are poorly understood. The study on *Leishmania amazonensis* infection confirmed that HIF-1α promotes survival of parasites but the mechanisms are largely unknown (Arrais-Silva et al., 2005). This study confirmed that *L. donovani* infection activates HIF-1α expression, which eventually alters the production of inflammatory cytokines in the favor of parasite survival through upregulation of miR-210 expression.

HypoxamiR is a class of miRNAs that is induced under influence of hypoxia in different types of primary and transformed cells (Devlin et al., 2011). MicroRNA-210 is the most important hypoxamiR, found in many cell types including macrophages. The HIF-1α binds to highly conserved sequences, i.e., hypoxia responsive element (HRE) on the proximal miR-210 promoter, which is located 400 bp upstream on the chromosome 11p15.5 (Huang et al., 2010). The miRNA profiles of macrophages have been done in response to different species of *Leishmania* infection (Lemaire et al., 2013; Geraci et al., 2015; Singh et al., 2016). In our present study, we found that during course of *L. donovani* infection, miR-210 expression was upregulated that helps in survival of parasites by suppression of host pro-inflammatory immune response. Further, the regulation of miR-210 expression in *Leishmania* infection was found to be HIF-1α dependent as silencing of HIF-1α, downregulated the miR-210 expression. This study suggests that *L. donovani* exploits activation of HIF-1α and miR-210 in mammalian host for its survival and growth. The findings of this study may open many lock and be useful in exploring immunological regulation during VL.

HIF-1α dependent activation of NF-κB has also been demonstrated in cancer cell lines (Bandarra et al., 2015). It is well established that HIF-1α directly or indirectly regulates NF-κB activation but the intermediate molecules between these two are not yet identified. Further, the activation of the transcription factor NF-κB in *L. donovani* infection is poorly understood (Guizani-Tabbane et al., 2004). In the present study, we found that miR-210 targets many genes of inflammatory immune response that was observed by online software miRDB. TNF receptor family is sixth rank target gene of miR-210 that was further validated by luciferase activity in our study and helps in NF-κB activation. TNF-α is one of the main inducers of NF-κB family and our results pointed its miR-210 mediated immune-regulation during VL that favors survival of parasites.

The NF-κB activated by the action of several stimuli such as inflammatory cytokines, bacterial products, viruses, physical and physiological stresses, and the pathway regulating TNF-α mediated NF-κB activation has been well established (Zhou et al., 2003). It has been previously reported that active NF-κB p50 forms heterodimer with p65 or c-Rel and translocates into nucleus where it binds to consensus sequences and activates the transcription of various genes (Chen and Ghosh, 1999). This study showed that inhibition of miR-210 by antagomir activates NF-κBp50 and augments the pro-inflammatory immune response. The NF-κBp50 has been shown to play an important role in pro-inflammatory immune responses (Tak and Firestein, 2001). In addition, it has also been shown to be involved in activation of inflammatory cytokine TNF-α in human monocytes by blocking the anti-inflammatory cytokine, i.e., IL-10 production (Schottelius et al., 1999). This study adds a new insight that TNF-α production is NF-κB dependent, which is broadly controlled by miR-210 during course of *L. donovani* infection.

After infection, *Leishmania* struggles for survival inside the macrophages that is directly linked to its combat with pro-oxidants like O_2_ and H_2_O_2_. After phagocytosis of parasites, bacteria, and other pathogens by a phagocytic cell, the ROS are rapidly produced by NADPH oxidase and help in the generation of anti-pathogenic environment (Horta et al., 2012). These toxic radicals, especially produced by macrophages and neutrophils, react with pathogens proteins, lipids, and nucleic acids, which eventually lead to their killing (Iles and Forman, 2002; Winyard et al., 2005). These radicals also augment the inflammatory response by playing the regulatory role of tyrosine and mitogen-activated protein kinases and activation of transcription factors like NF-κB, AP-1 leading to the production of pro- or anti-inflammatory cytokines (Closa and Folch-Puy, 2004). *Leishmania* species are known to downregulate the production of toxic free radicals that are considered as major macrophage effector molecules to control infection (Gantt et al., 2001). This study showed that HIF-1α induces miR-210 expression, which also controls respiratory burst activities of macrophages during *Leishmania* infection. After silencing of miR-210, the increased levels of NO*_x_* and ROS were observed that might be due to pro-inflammatory cytokines activation, which influenced the production of these free radicals and helped in the killing of parasites.

## Conclusion

To conclude, the HIF-1α helps *L. donovani* survival through mir-210 upregulation. Further, the upregulated miR-210 inhibits TNF-α receptor family leading to decreased synthesis of various pro-inflammatory cytokines, which helps the parasite survival inside the macrophages. However, after silencing the miR-210 with antagomir, TNF-α stimulates NF-κB p50, which forms heterodimer with subunit of NF-κB and translocates into the nucleus where it binds to consensus sequences and promotes transcription of pro-inflammatory cytokines genes. This cascade further augments the ROS and NO production leading to killing of parasites.

## Author Contributions

VK and AjK designed and performed the experiments. AsK, KA, SD, SV, and AM helped in the design of the study and statistical analysis. VK, RS, and PD designed and co-wrote the manuscript. PD conceived, designed, directed, and supervised the complete study.

## Conflict of Interest Statement

The authors declare that the research was conducted in the absence of any commercial or financial relationships that could be construed as a potential conflict of interest.
