# Supplementary material for: Leishmania donovani Activates Hypoxia Inducible Factor-1α and miR-210 for Survival in Macrophages by Downregulation of NF-κB Mediated Pro-inflammatory Immune Response
Source: Front Microbiol. 2018 Mar 8;9:385. doi: 10.3389/fmicb.2018.00385 (PMC5852103; doi:10.3389/fmicb.2018.00385)
Supplement: Supplementary file 1 [file Table_1.DOCX]

**Supplementary** **table 1:** List of primers used in the study.

| miRNA-Primers | |
| --- | --- |
| Forward Primers | Stem Loop Primers |
| **1.** **mmu-mir 202:**  5’ GTGCTGTACTGACTTGATGAAA 3’  **2.** **mmu-mir 210:** 5’ TTA GAA AGC CAC TGC CCA CC 3’  **Universal Reverse Primer**  **1.** 5’ GTGCAGGGTCCGAGG 3’ | **1.** 5’ GTT GGC TCT GGT GCA GGG TCC GAG GTA TTC GCA CCA GAG CCA ACC ATC AG 3’  **2.** 5’ GTC GTA TCC AGT GCA GGG TCC GAG GTA TTC GCA CTG GAT ACG AC  CAGTGT 3’ |
| **Cytokines Primer** | |
| Forward primer | ′Reverse primer |
| **TNF-α**  5′CCGATGGGTTGTACCTTGTC3  **IL-12:** 5′GGAGGACCCATAAGACTGC 3′  **IL-10:**5’ACCTGGTAGAAGTGATGCCCAGGCA3’  **β-actin**  5AACCGCGAGAAGATGACCCAGATCATGTTT3′  **′ HIF-1α**  **5’-**CCCATTCCTCATCCGTCAAATA-3’  **GAPDH**  5’ CAAGGCTGTGGGCAAGGTCA 3’ | 5’CGGACTCCGCAAAGTCTAAG3’  5′TTTCCCCTTCTTGGAGGTTT3  5’CTATGCAGTTGATGAAGATCGTCAAA3’  5’AGCAGCCGTGGCCATCTCTTGCTCGAAGTC-3’  5’-CCTGTGGTGACTTGTCCTTTAG-3’  5’AGGTGGAAGAGTGGGAGTTGCTG 3’ |
