# Supplementary material for: Leishmania donovani Activates Hypoxia Inducible Factor-1α and miR-210 for Survival in Macrophages by Downregulation of NF-κB Mediated Pro-inflammatory Immune Response
Source: Front Microbiol. 2018 Mar 8;9:385. doi: 10.3389/fmicb.2018.00385 (PMC5852103; doi:10.3389/fmicb.2018.00385)
Supplement: Supplementary file 2 [file Data_Sheet_1.pdf]

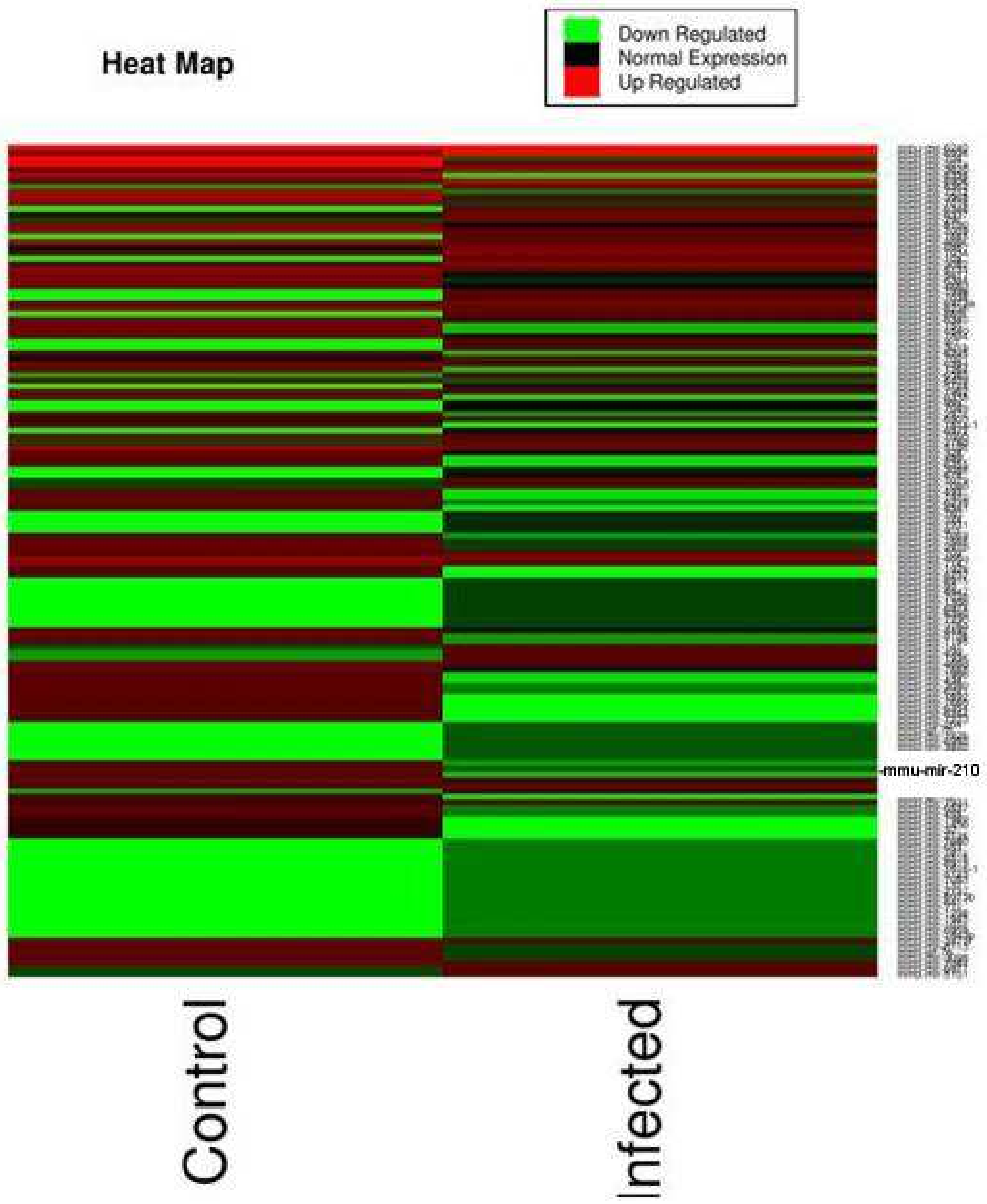

Supplementary material 1: Heat map of control and *L.donovani* infected macrophages

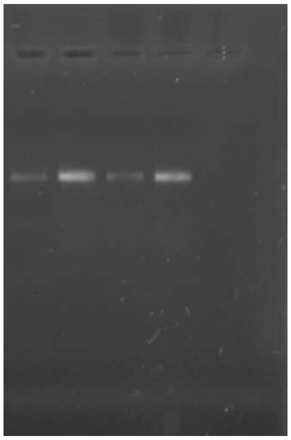

Supplementary Figure 2: Original PCR gel of HIF-1α

There are 346 predicted targets for mmu-miR-210-5p in miRDB.

Target Detail	Target Rank	Target Score	miRNA Name	Gene Symbol	Gene Description
Details	1	99	mmu-miR-210-5p	Akirin2	akirin 2
Details	2	98	mmu-miR-210-5p	Epg5	ectopic P-granules autophagy protein 5 homolog (C. elegans)
Details	3	98	mmu-miR-210-5p	Bach2	BTB and CNC homology 2
Details	4	98	mmu-miR-210-5p	Mtmr4	myotubularin related protein 4
Details	5	97	mmu-miR-210-5p	Tnrc6a	trinucleotide repeat containing 6a
Details	6	97	mmu-miR-210-5p	Tnfrsf21	tumor necrosis factor receptor superfamily, member 21
Details	7	96	mmu-miR-210-5p	Pdcd10	programmed cell death 10
Details	8	96	mmu-miR-210-5p	Mob3a	MOB kinase activator 3A
Details	9	95	mmu-miR-210-5p	Vgll3	vestigial like 3 (Drosophila)
Details	10	95	mmu-miR-210-5p	Reln	reelin
Details	11	94	mmu-miR-210-5p	Gars	glycyl-tRNA synthetase
Details	12	94	mmu-miR-210-5p	Gabra4	gamma-aminobutyric acid (GABA) A receptor, subunit alpha 4
Details	13	94	mmu-miR-210-5p	Arl11	ADP-ribosylation factor-like 11
Details	14	94	mmu-miR-210-5p	Fndc3a	fibronectin type III domain containing 3A
Details	15	94	mmu-miR-210-5p	2810403A07Rik	RIKEN cDNA 2810403A07 gene
Details	16	93	mmu-miR-210-5p	Rassf4	Ras association (RalGDS/AF-6) domain family member 4
Details	17	93	mmu-miR-210-5p	Fchsd2	FCH and double SH3 domains 2
Details	18	93	mmu-miR-210-5p	Ndst2	N-deacetylase/N-sulfotransferase (heparan glucosaminyl) 2
Details	19	93	mmu-miR-210-5p	Ddx3x	DEAD/H (Asp-Glu-Ala-Asp/His) box polypeptide 3, X-linked
Details	20	92	mmu-miR-210-5p	Serinc5	serine incorporator 5

Supplementary figure 3: List of Target gene of miRNA-210 by online software miRDB. Only top 20 ranked gene shown in the figure.

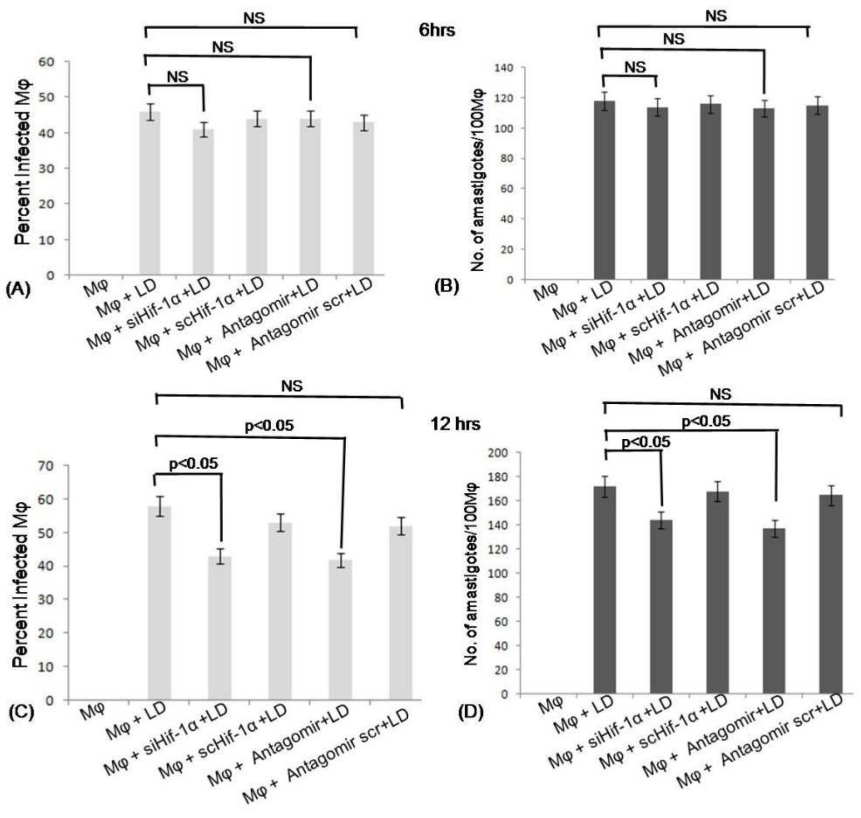

Supplementary figure 4: Percentage infectivity and parasite survival at the different time point.

4 A & B: The percentage infectivity and survival in siHIF-1α or antagomir-210 treated macrophages after 6hrs of infection. **4 C & D:** The percentage infectivity and survival in siHIF-1α or antagomir-210 treated macrophages after 12 hrs of infection. we observed that after 6 h of infection, the percentage infectivity and parasite survival did not change significantly in siHIF-1α or antagomir-210 treated macrophages however after 12 h of infection in siHIF-1α or antagomir-210 treated macrophages the percentage infectivity and amastigotes number per 100 macrophages was significantly ($p<0.05$) reduced.

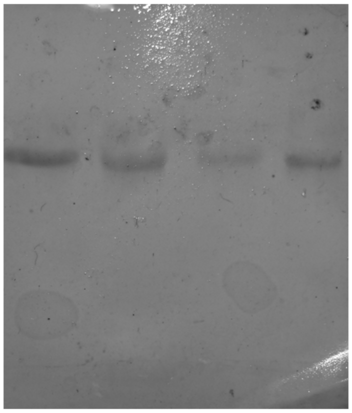

Supplementary figure 5: Original image of Western blot for figure 8.A

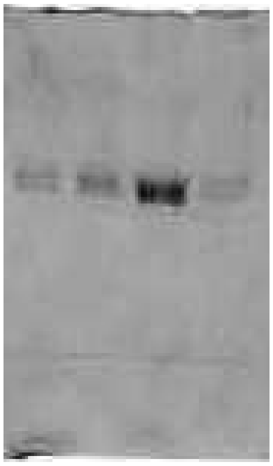

Supplementary figure 6: Original image of Western blot for figure 8.B

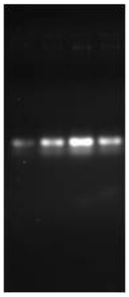

Supplementary figure 7. Original image of semi-quantitative PCR of TNF-α for Figure 9.

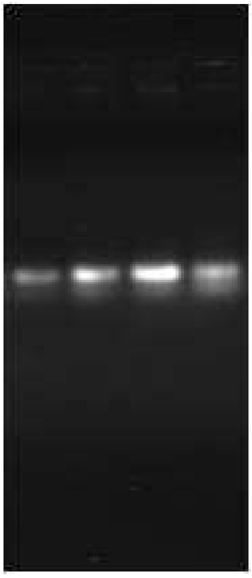

Supplementary figure 8. Original image of semi-quantitative PCR of IL-12 for Figure 9.

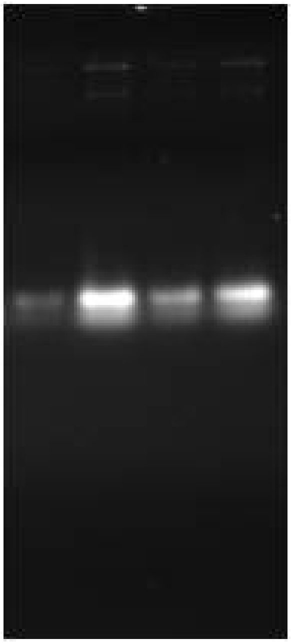

Supplementary figure 9. Original image of semi-quantitative PCR of IL-10 for Figure 9
